# Supplementary material for: Cornering the universal shape of fluctuations
Source: Nat Commun. 2022 Jan 12;13:287. doi: 10.1038/s41467-021-27727-1 (PMC8755810; doi:10.1038/s41467-021-27727-1)
Supplement: Supplementary file 1 — Supplementary Information [file 41467_2021_27727_MOESM1_ESM.pdf]

Supplementary Information for: Cornering the universal shape of fluctuations

Benoit Estienne,[1] Jean-Marie Stéphan,[2] and William Witczak-Krempa[3,4,5]

[1]*Sorbonne Université, CNRS, Laboratoire de Physique Théorique et Hautes Énergies, LPTHE, F-75005 Paris, France.*
[2]*Univ Lyon, CNRS, Université Claude Bernard Lyon 1, UMR5208, Institut Camille Jordan, F-69622 Villeurbanne, France*
[3]*Département de Physique, Université de Montréal, Montréal, Québec, H3C 3J7, Canada*
[4]*Centre de Recherches Mathématiques, Université de Montréal; P.O. Box 6128, Centre-ville Station; Montréal (Québec), H3C 3J7, Canada*
[5]*Regroupement Québécois sur les Matériaux de Pointe (RQMP)*

SUPPLEMENTARY NOTE 1: VOLUME AND AREA LAWS IN ANY DIMENSION

In this appendix, we compute explicitly the volume and area law coefficients in any dimension d. While such scaling is well known for the fluctuations, we report here some general formulas in terms of the connected correlation function f. Our starting point is Eq.(5) in the main text

$$(\Delta\mathcal{O}_A)^2 = \alpha|A| + \Theta_A \tag{1}$$

where

$$\alpha = \int d^d\mathbf{r} f(\mathbf{r}) = \frac{2\pi^{\frac{d}{2}}}{d\,\Gamma\left(\frac{d}{2}\right)}\int_0^\infty dr\, r^{d-1} f(r) \tag{2}$$

is the coefficient of the volume term, the remaining term

$$\Theta_A = -\int_A d^d\mathbf{r}_1 \int_{A^c} d^d\mathbf{r}_2\, f(|\boldsymbol{r}_1 - \boldsymbol{r}_2|) \tag{3}$$

scales with the size of the boundary, $|\partial A|$, for large A:

$$\Theta_A = \beta|\partial A| + \cdots \tag{4}$$

where the ellipsis denote subleading terms. Intuitively this scaling comes from the fact that the previous integral is dominated by the region close to the interface between A and A^c, provided f decays reasonably fast. To get an explicit formula for β, we first transform the double integral over A and A^c into a double boundary integral over ∂A using the following relation

$$\Theta_A = -\int_{\partial A} d\sigma_1 \int_{\partial A} d\sigma_2\, (\mathbf{n}_1\cdot\mathbf{n}_2)\, F(|\mathbf{r}_1 - \mathbf{r}_2|) \tag{5}$$

where $\boldsymbol{n}_1, \boldsymbol{n}_2$ are unit vectors normal to the boundary of A, and $F(r)$ is such that its laplacian satisfies $\Delta F = f$, that is $\partial_r(r^{d-1}\partial_r F(r)) = r^{d-1} f(r)$. We now pick a specific geometry where calculations are simple. In $\mathbb{R}^d$ we take for A the half-space $x_d \geq 0$, with boundary ∂A being the hyperplane $x_d = 0$.

$$\Theta_A = -\int_{\mathbb{R}^{d-1}} d^{d-1}\mathbf{r}_1 \int_{\mathbb{R}^{d-1}} d^{d-1}\mathbf{r}_2\, F(|\mathbf{r}_1 - \mathbf{r}_2|) = |\partial A| \int_{\mathbb{R}^{d-1}} d^{d-1}\mathbf{r}\, F(|\mathbf{r}|) \tag{6}$$

where we changed variable to the center of mass $\mathbf{r} = \mathbf{r}_1 - \mathbf{r}_2$. The fact that $|\partial A|$ is infinite is not really an issue, as one can repeat the same argument in finite volume (*e.g.* working in a box with periodic boundary conditions). Thus

$$\beta = -\int_{\mathbb{R}^{d-1}} d^{d-1}\mathbf{r}\, F(|\mathbf{r}|) = -\frac{2\pi^{\frac{d-1}{2}}}{\Gamma\left(\frac{d-1}{2}\right)}\int_0^\infty r^{d-2} F(r) \tag{7}$$

Integrating by parts twice yields

$$\beta = -\frac{\pi^{\frac{d-1}{2}}}{\Gamma\left(\frac{d+1}{2}\right)} \int_0^\infty dr\, r^d f(r) \tag{8}$$

It is important to stress that while the computation of the area law coefficient β has been done in this simple geometry, the result holds irrespective of the precise shape of the boundary, unless f decays too slowly. Upon rescaling the region $A \to LA$, the fluctuations behaves for large L as

$$(\Delta\mathcal{O}_{LA})^2 \sim \alpha L^d |A| + \beta L^{d-1} |\partial A| + \cdots \tag{9}$$

In this asymptotic regime, the boundary can be locally approximated by its tangent hyperplane, for which our computation applies.

As a side note, in two dimensions it is rather suggestive that the coefficients of the volume, boundary, and corner terms are respectively proportional to

$$\int_0^\infty dr\, r f(r), \qquad \int_0^\infty dr\, r^2 f(r) \quad \text{and} \quad \int_0^\infty dr\, r^3 f(r)\,. \tag{10}$$

Whether this remarkable sequence extends into higher dimensions is an interesting question.

SUPPLEMENTARY NOTE 2: TWO DERIVATIONS OF THE SUPER-UNIVERSAL BEHAVIOR

We provide further information regarding the derivation of our main result, Eq. (4) of the main text. We first present the computation of the remaining four-dimensional integral, which gives the angular dependence of the corner function. We also present an independent alternative derivation of the super-universal corner function.

The remaining integral

In this appendix we evaluate the integral

$$b(\theta) = -\int_B d\mathbf{r_1} \int_D d\mathbf{r_2} f(|\mathbf{r_1} - \mathbf{r_2}|) \tag{11}$$

This can be done as follows. We first rewrite

$$\int_B d\mathbf{r_1} \int_D d\mathbf{r_2} f(|\mathbf{r_1} - \mathbf{r_2}|) = \int_0^\infty dr f(r) \rho(r,\theta), \qquad \text{where} \qquad \rho(r,\theta) = \int_B d\mathbf{r_1} \int_D d\mathbf{r_2} \delta(|\mathbf{r_1} - \mathbf{r_2}| - r) \tag{12}$$

The point is now that the regions B and D being cones, they are invariant under dilatations. Rescaling $r_i \to r r_i$ thus yields

$$\rho(r,\theta) = \int_B d\mathbf{r_1} \int_D d\mathbf{r_2} \delta(|\mathbf{r_1} - \mathbf{r_2}| - r) = r^3 \rho(1,\theta) \tag{13}$$

and we obtain the factorization of the angular and radial variables

$$b(\theta) = -\rho(1,\theta) \int_0^\infty \frac{r^3}{2} f(r)\, dr\,. \tag{14}$$

Strikingly the angular function $\rho(1,\theta)$ does not depend on the connected density-density two-point function. The angular dependence can be computed [1], yielding for $\theta \in [0, 2\pi]$

$$b(\theta) = -\left(1 + (\pi - \theta)\cot\theta\right) \int_0^\infty \frac{r^3}{2} f(r)\, dr\,. \tag{15}$$

An alternative derivation

For completeness, we present here an alternative derivation of our main result. Our main goal is to isolate the corner function from the dominant volume and area law term. This can be done in a different way, simply noticing that those terms are affine in θ, so can be eliminated by differentiating twice with respect to θ. Denoting by $D_2(\theta)$ this second derivative, we have

$$D_2(\theta) = \frac{d^2}{d\theta^2}\int_A d\mathbf{r}_1 \int_A d\mathbf{r}_2 f(|\mathbf{r}_1 - \mathbf{r}_2|) \tag{16}$$

$$= \frac{d^2}{d\theta^2}\int_0^R r_1 dr_1 \int_0^\theta d\theta_1 \int_0^R r_2 dr_2 \int_0^\theta d\theta_2 f(\sqrt{r_1^2 + r_2^2 - 2r_1 r_2 \cos(\theta_1 - \theta_2)}) \tag{17}$$

where we integrate on an angular sector of a finite disk with radius R for now. Using the identity $\frac{d^2}{d\theta^2}\int_0^\theta d\theta_1 \int_0^\theta d\theta_2 g(\theta_1 - \theta_2) = g(\theta) + g(-\theta)$ and sending R to infinity yields

$$D_2(\theta) = 2\int_0^\infty r_1 dr_1 \int_0^\infty r_2 dr_2 f(\sqrt{r_1^2 + r_2^2 - 2r_1 r_2 \cos\theta}) \tag{18}$$

which is finite. This can be evaluated by seeing r_1 and r_2 as cartesian coordinates, and switching to polar variables

$$D_2(\theta) = \int_0^{\pi/2} d\omega \sin(2\omega) \int_0^\infty \rho^3 d\rho f(\rho\sqrt{1 - \sin 2\omega \cos\theta}). \tag{19}$$

Finally rescaling ρ, we obtain

$$D_2(\theta) = \int_0^{\pi/2} \frac{d\omega \sin 2\omega}{(1 - \sin 2\omega \cos\theta)^2} \int_0^\infty d\rho \rho^3 f(\rho) \tag{20}$$

$$= \frac{1 + (\pi - \theta)\cot\theta}{\sin^2\theta} \int_0^\infty d\rho \rho^3 f(\rho). \tag{21}$$

Integrating twice, the integration constants may be set by requiring $b(\pi) = 0$ and $b(2\pi - \theta) = b(\theta)$, and we recover Eq. (4) of the main text.

SUPPLEMENTARY NOTE 3: ANALYTIC COMPUTATION OF THE CORNER TERM FOR THE INTEGER QUANTUM HALL EFFECT

In this appendix we compute the connected two-point function $f(r)$ for the $\nu = n$ integer quantum Hall effect. We obtain

$$f(r) = \frac{n}{2\pi l_B^2}\delta(\boldsymbol{r}) - \frac{1}{4\pi^2 l_B^4} e^{-\frac{r^2}{2l_B^2}} \left(L_{n-1}^{(1)}\left(\frac{r^2}{2l_B^2}\right)\right)^2, \tag{22}$$

where l_B is the magnetic length and $L_{n-1}^{(1)}$ is the associated Laguerre polynomial. One can readily check that the volume term vanishes, as expected from particle number conservation. The sum rule in Eq. (4) can then be computed exactly, yielding

$$\int_0^\infty \frac{r^3}{2} f(r) dr = -\frac{n}{4\pi^2}. \tag{23}$$

In order to derive the above relation, we first note that the corner contribution does not depend on the magnetic length l_B by virtue of being dimensionless. Thus without loss of generality we set $l_B = 1$. The pth Landau level is

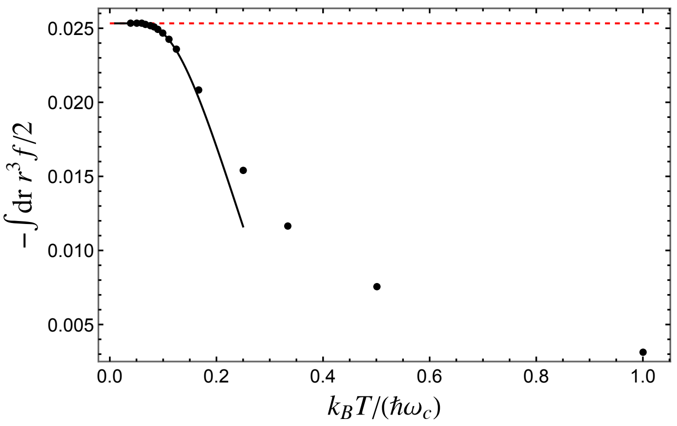

Supplementary Figure 1. Corner coefficient of the integer quantum Hall effect at finite temperature. Temperature dependence of the corner coefficient for charge fluctuations at filling $\nu = 1$. The red dashed line is the $T = 0$ result, $1/(4\pi^2)$. The solid line shows the small temperature expansion, Supplementary Eq. (30).

spanned by the states $|p, m\rangle$ with wavefunctions (in the symmetric gauge)

$$\Psi_{p,m}(z) = \frac{\sqrt{p!}}{\sqrt{2^{m-p} m!}} \frac{1}{\sqrt{2\pi}} z^{m-p} L_p^{(m-p)} \left(\frac{z\bar{z}}{2}\right) e^{-\frac{z\bar{z}}{4}} \tag{24}$$

where $z = x + iy$ and the integer m ranges over all non negative integers. The integer quantum Hall effect at filling fraction $\nu = n$ is obtained by occupying all Landau levels from $p = 0$ to $p = n - 1$. For such a non-interacting fermionic system, the connected density-density two-point function can be computed via Wick's theorem

$$\langle \rho(\mathbf{r_1})\rho(\mathbf{r_2})\rangle_c = \langle \rho(\mathbf{r_2})\rangle\, \delta(\mathbf{r_1} - \mathbf{r_2}) - |K(\mathbf{r_1}, \mathbf{r_2})|^2 = K(\mathbf{r_1}, \mathbf{r_1})\, \delta(\mathbf{r_1} - \mathbf{r_2}) - |K(\mathbf{r_1}, \mathbf{r_2})|^2 \tag{25}$$

where $K(\mathbf{r_1}, \mathbf{r_2}) = \langle \Psi^\dagger(\mathbf{r_1})\Psi(\mathbf{r_2})\rangle$ is the kernel of the projector onto the occupied states. At filling $\nu = n$, this is

$$K(\mathbf{r_1}, \mathbf{r_2}) = \sum_{p=0}^{n-1} \sum_{m=0}^{\infty} \Psi_{p,m}(z_1)\overline{\Psi_{p,m}(z_2)} = \frac{1}{2\pi} e^{\frac{z_1\bar{z}_2}{2}} e^{-\frac{z_1\bar{z}_1 + z_2\bar{z}_2}{4}} L_{n-1}^{(1)}\left(\frac{|z_1 - z_2|^2}{2}\right) \tag{26}$$

where $z_j = x_j + iy_j$, yielding Supplementary Eq. (22).

If we entirely fill only the nth Landau level for some $n \geq 0$, and leave all other levels empty, the kernel is modified to

$$K(\mathbf{r_1}, \mathbf{r_2}) = \sum_{m=0}^{\infty} \Psi_{n,m}(z_1)\overline{\Psi_{n,m}(z_2)} = \frac{1}{2\pi} e^{\frac{z_1\bar{z}_2}{2}} e^{-\frac{z_1\bar{z}_1 + z_2\bar{z}_2}{4}} L_{n}^{(0)}\left(\frac{|z_1 - z_2|^2}{2}\right) \tag{27}$$

and we find

$$\int_0^\infty \frac{r^3}{2} f(r) dr = -\frac{2n+1}{4\pi^2}\,. \tag{28}$$

Fluctuations at finite temperature

The preceding discussion was concerned with eigenstates of the IQH Hamiltonian. We now analyze the charge fluctuations in IQH states at finite temperature T. We work in the grand canonical ensemble, where the chemical potential μ is determined by requiring that the average density be an integer $\nu \in \{1, 2, 3, \cdots\}$. The implicit equation for the chemical potential can be numerically solved for a given temperature. The next step is to compute the kernel

K, which then determines the density-density correlation function through Supplementary Eq. (25). Explicitly, we have for $T \geq 0$

$$K(\mathbf{r},0) = \langle \Psi^\dagger(\mathbf{r})\Psi(0)\rangle_T = \frac{\exp(-r^2/4)}{2\pi}\sum_{n=0}^{\infty} n_F(\xi_n) L_n^{(0)}(r^2/2) \tag{29}$$

where $\xi_n = E_n - \mu$ is the LL energy shifted by the temperature-dependent chemical potential and n_F stands for the Fermi-Dirac distribution. It can be shown that the tail of the series in Supplementary Eq. (29) contributes an exponentially decaying function of the separation r. This implies that charge-charge correlation function f decays exponentially with r, and the super-universal shape dependence holds, with the prefactor given in Eq. (4) of the main text. The full dependence of the prefactor can be numerically calculated, we show the result in Supplementary Figure 1. In particular, at low T, we find that it remains unchanged up to corrections that are exponentially small in the ratio of the cyclotron energy to twice the thermal energy, $\hbar\omega_c/(2k_BT)$:

$$-\int_0^\infty dr\, \frac{r^3}{2} f(r) = \frac{\nu}{4\pi^2}\left[1 - 4\exp(-\hbar\omega_c/(2k_BT))\right] + \cdots \tag{30}$$

where the ellipsis denote subleading terms in the small T limit. Such a suppression is natural due to the cyclotron gap.

A new property that arises at finite temperature for charge fluctuations is a volume law term, which vanishes in the groundstate due to charge conservation. The coefficient of the volume law α is given by Supplementary Eq. (2). Using the orthonormality of Laguerre polynomials, we find

$$\alpha = \frac{\nu}{2\pi} - \frac{1}{2\pi}\sum_{n=0}^{\infty} n_F(\xi_n)^2 \tag{31}$$

At low temperature, and unit filling, we find

$$\alpha = \frac{1}{\pi}\exp(-\hbar\omega_c/(2k_BT)) + \cdots \tag{32}$$

which decays exponentially fast as $T \to 0$.

SUPPLEMENTARY NOTE 4: CONFORMAL FIELD THEORIES

For conserved charge correlation functions of CFTs in d spatial dimensions, the universal large distance behavior of the correlation function is $f(r) \sim r^{-2d}$. The leading terms of the fluctuations ΔO_A^2 for large regions A are dominated by this infra-red behavior, including the corner terms. Thus we can ignore the short-distance behavior of $f(r)$ in evaluating ΔO_A^2, at the cost of introducing a short-distance cut-off. We can transform the double integral over A into a double boundary integral over ∂A by virtue of the following relation:

$$\int_A d\mathbf{r}_1 \int_A d\mathbf{r}_2\, \frac{1}{|\mathbf{r}_1 - \mathbf{r}_2|^{2d}} = -\frac{1}{2d(d-1)}\int_{\partial A} d\sigma_1 \int_{\partial A} d\sigma_2\, \frac{\mathbf{n}_1\cdot\mathbf{n}_2}{|\mathbf{r}_1-\mathbf{r}_2|^{2(d-1)}} \tag{33}$$

where $\mathbf{n}_1, \mathbf{n}_2$ are unit vectors normal to the boundary of A. Note the *important minus sign* on the RHS. Subtleties can arise due to the short-distance divergent nature of both sides, but these can be taken care of via a short-distance regulator and do not affect the universal coefficients that interest us. Supplementary Eq. (33) can be shown by starting with the r.h.s., and using Stokes theorem twice. The r.h.s. of Supplementary Eq. (33) is seen to be exactly the form of the Extensive Mutual Information (EMI) model for the entanglement entropy [2–4]. In $d = 2$ spatial dimensions, the integral for region A being a corner of angle θ has been computed in numerous references [3–5]. The answer is:

$$\int_{\partial A} d\sigma_1 \int_{\partial A} d\sigma_2\, \frac{\mathbf{n}_1\cdot\mathbf{n}_2}{|\mathbf{r}_1-\mathbf{r}_2|^2} = B\frac{L}{\delta} - a(\theta)\ln(L/\delta) + \cdots \tag{34}$$

where $B > 0$ is the non-universal coefficient of the boundary law, and the EMI corner term reads

$$a(\theta) = 2(1 + (\pi - \theta)\cot\theta) \tag{35}$$

This leads to the universal charge fluctuation corner function of CFTs given in the main text. It is important to note that this serves as an independent derivation of our result for $b(\theta)$, Eq. (4) of the main text, for the case when $f(r) \sim 1/r^4$ at large distances.

In addition, our relation between bipartite fluctuations and the EMI for entanglement entropy gives a concrete realization of the latter, and allows to use many of the results previously obtained in a new context. We note that a relation similar, but distinct to Supplementary Eq. (34) was previously obtained in Ref. 6.

SUPPLEMENTARY NOTE 5: SLOW DECAY AND METALS

In this appendix, we focus on two-point functions that decay slower than the CFT one for a conserved charge density, which leads to different behavior for bipartite fluctuations. In particular, it is not possible anymore to interpret the term $b(\theta)$ as a corner contribution: as we shall see, this term will depend on the whole shape of region A. For concreteness, we consider a two-point function decaying as

$$f(r) \sim \frac{a}{r^{4h}} \tag{36}$$

for large r, and exponent $3/4 \leq h < 1$. For region A we take a circular sector with radius L and angle θ. We have

$$(\Delta\mathcal{O}_A)^2 = \int_0^L r_1 dr_1 \int_0^L r_2 dr_2 \int_0^\theta d\theta_1 \int_0^\theta d\theta_2 f(\sqrt{r_1^2 + r_2^2 - 2r_1 r_2 \cos(\theta_1 - \theta_2)}) \tag{37}$$

To try and indentify an analog of the corner contribution, we define $D_2(\theta)$ as the second derivative of the variance. Using polar coordinates $r_1 = \rho\cos\omega$, $r_2 = \rho\sin\omega$, it may be expressed as

$$D_2(\theta) = \int_0^{\pi/2} d\omega \sin 2\omega \int_0^{L/\cos\left(\frac{\pi}{4} - |\omega - \frac{\pi}{4}|\right)} d\rho\, \rho^3 f(\rho\sqrt{1 - \sin 2\omega\cos\theta}) \tag{38}$$

$$\sim \frac{aL^{4-4h}}{2(1-h)} \int_0^{\pi/4} d\omega \frac{\sin 2\omega [\cos\omega]^{4h-4}}{[1 - \sin 2\omega \cos\theta]^{2h}} \tag{39}$$

Hence, the (second derivative of the) "corner term" diverges as a power law in L. The angular dependence is no longer the super-universal function, because of the extra factor $(\cos\omega)^{4h-4}$, and the change in exponent in the denominator. The former can be traced back to the exterior (circular) boundary of A, which now enters the calculation due to the long-range correlation. We stress that the θ dependent correction we compute here should not be interpreted as a corner term. Indeed it is sensitive to the shape of A as a whole. For instance modifying the region A even very far from the corner, such as changing the exterior boundary, does affect the angular dependence. Nevertheless we can consider the divergence of this correction term for $\theta \to 0$. It can be computed by noticing that the integral is dominated by the vicinity of $\omega = \pi/4$, in which case the denominator blows up. Expanding in ω up to distances of order $\sqrt{\theta}$ yields the estimate

$$\int_0^{\pi/4} d\omega \frac{\sin 2\omega [\cos\omega]^{4h-4}}{[1 - \sin 2\omega\cos\theta]^{2h}} \underset{\theta\to 0}{\sim} \frac{\sqrt{\pi}\,\Gamma(2h - 1/2)}{\Gamma(2h)} \theta^{1-4h}, \tag{40}$$

where Γ is the usual Gamma function. We also checked that the method combining four corners, which is explained in the main text, gives the same result. Integrating twice the last integral yields a divergence as θ^{3-4h} for $h \in (3/4, 1)$, and $\log\theta$ for $h = 3/4$. This is to be compared with the $1/\theta$ divergence valid for any $h \geq 1$.

Let us finally discuss the case of metals. For an isotropic Fermi sea $|\mathbf{k}| < k_F$, the non-interacting fermion propagator is

$$\langle \Psi^\dagger(\mathbf{r_1})\Psi(\mathbf{r_2})\rangle = K(\mathbf{r_1}, \mathbf{r_2}) = \int_{|\mathbf{k}|<k_F} \frac{d\mathbf{k}}{(2\pi)^2} e^{i\mathbf{k}\cdot(\mathbf{r_1}-\mathbf{r_2})} = \frac{k_F}{2\pi r} J_1(rk_F) \tag{41}$$

where $r = |\mathbf{r_1} - \mathbf{r_2}|$, and $J_1(z)$ is the Bessel function of the first kind. Therefore the connected two-point function is

$$f(r) = \frac{k_F^2}{4\pi}\frac{\delta(r)}{2\pi r} - \frac{k_F^2}{4\pi^2 r^2} J_1^2(rk_F) \tag{42}$$

and up to the usual oscillations behaves at large distances as $f(r) \sim ar^{-3}$ for some constant a. In momentum space, this translates to the scaling $S(k) \propto k$ at small k, where $S(k)$ is the spatial Fourier transform of f. This scaling fits in the above discussion, with exponent $h = 3/4$. For an interacting Fermi liquid (FL), the same scaling holds, but with a prefactor modified by the Landau parameter F_{0s}, as is discussed in Ref. 7 in the context of bipartite fluctuations. Therefore, for a FL $b(\theta)$ is of order L, $b(\theta) = L\,\mathfrak{b}_{\rm FL}(\theta)$. Recall the leading term of the variance corresponds to a logarithmically enhanced area law (given here for non-interacting fermions) [8]

$$(\Delta N_A)^2 \sim \frac{(2+\theta)}{2\pi^3} k_F L \log L\,, \tag{43}$$

and this term is, indeed, eliminated by differentiating twice. The integral in Supplementary Eq. (39) can be simplified. Integrating twice, there is an ambiguity in fixing the integration constants, since changing the cutoffs—that is e.g. changing $\log L \to \log(L/\epsilon)$ in Supplementary Eq. (43)—would result in an extra contribution of order L, which is affine in θ. Choosing the cutoffs by asking that $\mathfrak{b}_{\rm FL}(\theta) = \alpha_{\rm FL}\cdot(\theta-\pi)^2$ close to $\theta = \pi$, we obtain

$$\frac{\mathfrak{b}_{\rm FL}(\theta)}{8\alpha_{\rm FL}} = \log 4 - 4C - 2\log\left[\sin\frac{\theta}{2}\left(1+\sin\frac{\theta}{2}\right)\right] + (2\pi-\theta)\log\tan\frac{\theta}{4} + 4\,\mathrm{Im}\,\mathrm{Li}_2\left(i\cot\frac{\theta}{4}\right). \tag{44}$$

As stated above, this result is valid up to affine terms in θ. Here, C is Catalan's constant and Li_2 denotes the dilogarithm. To some extent this functional form can be seen as an analog of the corner term discussed in the text, although one should keep in mind that it is sensitive to the global geometry of the region A.

SUPPLEMENTARY REFERENCES

[1] Estienne, B. & Stéphan, J.-M. Entanglement spectroscopy of chiral edge modes in the quantum Hall effect. *Phys. Rev. B* **101**, 115136 (2020). 1911.10125.

[2] Casini, H., Fosco, C. D. & Huerta, M. Entanglement and alpha entropies for a massive Dirac field in two dimensions. *J. Stat. Mech.* **0507**, P07007 (2005). cond-mat/0505563.

[3] Casini, H. & Huerta, M. Remarks on the entanglement entropy for disconnected regions. *JHEP* **03**, 048 (2009). 0812.1773.

[4] Swingle, B. Mutual information and the structure of entanglement in quantum field theory (2010). 1010.4038.

[5] Bueno, P., Myers, R. C. & Witczak-Krempa, W. Universality of Corner Entanglement in Conformal Field Theories. *Phys. Rev. Lett.* **115**, 021602 (2015). 1505.04804.

[6] Herviou, L., Le Hur, K. & Mora, C. Bipartite fluctuations and topology of dirac and weyl systems. *Phys. Rev. B* **99**, 075133 (2019). URL https://link.aps.org/doi/10.1103/PhysRevB.99.075133.

[7] Swingle, B. & Senthil, T. Universal crossovers between entanglement entropy and thermal entropy. *Phys. Rev. B* **87**, 045123 (2013). URL https://link.aps.org/doi/10.1103/PhysRevB.87.045123.

[8] Gioev, D. & Klich, I. Entanglement entropy of fermions in any dimension and the widom conjecture. *Physical Review Letters* **96** (2006). URL http://dx.doi.org/10.1103/PhysRevLett.96.100503.
